# Comparison of Karyotypes in Two Hybridizing Passerine Species: Conserved Chromosomal Structure but Divergence in Centromeric Repeats

**DOI:** 10.3389/fgene.2021.768987

**Published:** 2021-12-06

**Authors:** Manon Poignet, Martina Johnson Pokorná, Marie Altmanová, Zuzana Majtánová, Dmitry Dedukh, Tomáš Albrecht, Jiří Reif, Tomasz S. Osiejuk, Radka Reifová

**Affiliations:** ^1^ Department of Zoology, Faculty of Science, Charles University, Prague, Czech Republic; ^2^ Department of Ecology, Faculty of Science, Charles University, Prague, Czech Republic; ^3^ Institute of Animal Physiology and Genetics, Czech Academy of Sciences, Liběchov, Czech Republic; ^4^ Institute of Vertebrate Biology, Czech Academy of Sciences, Brno, Czech Republic; ^5^ Institute for Environmental Studies, Faculty of Science, Charles University, Prague, Czech Republic; ^6^ Department of Zoology and Laboratory of Ornithology, Faculty of Science, Palacký University, Olomouc, Czech Republic; ^7^ Department of Behavioural Ecology, Institute of Environmental Biology, Faculty of Biology, Adam Mickiewicz University, Poznań, Poland

**Keywords:** chromosomal structure, karyotype evolution, comparative genomic hybridization, rDNA, centromere, GRC, birds, *Luscinia*

## Abstract

Changes in chromosomal structure involving chromosomal rearrangements or copy number variation of specific sequences can play an important role in speciation. Here, we explored the chromosomal structure of two hybridizing passerine species; the common nightingale (*Luscinia megarhynchos*) and the thrush nightingale (*Luscinia luscinia*), using conventional cytogenetic approaches, immunostaining of meiotic chromosomes, fluorescence *in situ* hybridization as well as comparative genomic hybridization (CGH). We found that the two nightingale species show conserved karyotypes with the same diploid chromosome number of 2n = 84. In addition to standard chromosomes, both species possessed a small germline restricted chromosome of similar size as a microchromosome. Just a few subtle changes in chromosome morphology were observed between the species, suggesting that only a limited number of chromosomal rearrangements occurred after the species divergence. The interspecific CGH experiment suggested that the two nightingale species might have diverged in centromeric repetitive sequences in most macro- and microchromosomes. In addition, some chromosomes showed changes in copy number of centromeric repeats between the species. The observation of very similar karyotypes in the two nightingale species is consistent with a generally slow rate of karyotype evolution in birds. The divergence of centromeric sequences between the two species could theoretically cause meiotic drive or reduced fertility in interspecific hybrids. Nevertheless, further studies are needed to evaluate the potential role of chromosomal structural variations in nightingale speciation.

## Introduction

Despite an increasing number of sequenced avian genomes (Jarvis et al., 2014; Zhang et al., 2014; Feng et al., 2020), we still know relatively little about the organization of the genomes into chromosomes and to what degree the chromosomal structure (i.e., number, size and collinearity of chromosomes) varies among species. It has been proposed that changes in chromosomal structure, including chromosomal translocations, inversions and copy number variations, may play an important role in the origin of reproductive isolation between species (White, 1978; Rieseberg, 2001; Wellenreuther et al., 2019; Zhang et al., 2021). For example, chromosomal translocations may cause problems with chromosome pairing, recombination and segregation during meiosis, which can lead to hybrid sterility (White, 1978; King, 1993; Homolka et al., 2007). Structural changes, such as inversions, may facilitate speciation by reducing the recombination rate within the structural variant, which may help to maintain the species-specific traits in the face of gene flow (Rieseberg, 2001; Ortíz-Barrientos et al., 2002; Butlin, 2005; Hoffmann and Rieseberg, 2008). Finally, copy number variations may serve as a source of adaptive phenotypic variation (Perry et al., 2007; Zhou et al., 2011; Iskow et al., 2012; Bruders et al., 2020; Minias et al., 2020) and, in the case of copy number variation of the centromeric repeats, they can affect chromosome segregation during meiotic division (Akera et al., 2019), which may, in the extreme case, cause sterility in hybrids (Hurst and Pomiankowski, 1991; Phadnis and Orr, 2009; Zhang et al., 2015). Despite the assumed importance of structural variants in speciation, there are still relatively few studies comparing chromosomal structure between closely related species in the early stages of divergence (Hooper and Price, 2015, 2017; Hooper et al., 2019; Weissensteiner et al., 2020).

Among terrestrial vertebrates, birds have relatively stable karyotypes, usually composed of approximately 40 pairs of chromosomes, which include around 10 macrochromosomes and 30 mostly indistinguishable microchromosomes (Christidis, 1990; Pichugin et al., 2001; Masabanda et al., 2004; Griffin et al., 2007; Ellegren, 2010; Nanda et al., 2011; Degrandi et al., 2020a). In addition to size, macrochromosomes and microchromosomes differ in their GC content, gene density, recombination rate and substitution rate (Auer et al., 1987; Rodionov et al., 1992; Smith et al., 2000; Burt, 2002; Axelsson et al., 2005). Although the number and size of chromosomes is quite conserved in birds, indicating that interchromosomal rearrangements are rare in this group, intrachromosomal rearrangements such as inversions can occur relatively frequently (Aslam et al., 2010; Völker et al., 2010; Ellegren, 2013; Hooper and Price, 2017; Rodrigues et al., 2017). There is also evidence for relatively frequent copy number variations among birds (Skinner et al., 2014). All birds possess a ZW sex determination system (ZZ for male; ZW for female) with a large Z chromosome and usually smaller heterochromatic W chromosome (dos Santos et al., 2015; Schartl et al., 2016; Barcellos et al., 2019). In addition, it has been revealed that passerines possess an additional chromosome in their germ cells, the so-called germ-line restricted chromosome (GRC) (Pigozzi and Solari, 1998; Kinsella et al., 2019; Torgasheva et al., 2019). This chromosome is eliminated from the somatic cells during early development; being maintained only in the germline. In some passerines the GRC represents a big macrochromosome, while in others, a small microchromosome (Torgasheva et al., 2019). However, the size of this chromosome has only been characterized in 16 species so far and it is not clear how often it differs among closely related species (Torgasheva et al., 2019).

To date, somatic karyotypes of approximately 1,000 avian species (i.e., 10% of all bird species) have been described using mostly classical cytogenetic techniques such as G- and C- banding and Giemsa staining (reviewed in Degrandi et al., 2020a). Such techniques enable rough estimation of the diploid chromosome number as well as the detection of large chromosomal translocations or inversions. However, distinguishing smaller-scale chromosomal rearrangements and counting the number of microchromosomes is usually challenging. Moreover, karyotypes from somatic cells do not allow for the detection of the GRC. The development of molecular cytogenetic methods, such as fluorescence *in situ* hybridization (FISH) and whole chromosome probes (Griffin et al., 1999), made more detailed cross-species comparisons of chromosomal structure possible, but have so far only been applied to relatively few avian species, with chicken probes mostly being used as a reference (reviewed in Kretschmer et al., 2018; Degrandi et al., 2020a). In addition, immunostaining of the synapsed chromosomes during meiosis provides a useful approach for detection of the GRC and comparing the chromosomal structure among species (Hale et al., 1988; Torgasheva et al., 2019).

Based on the FISH technique Kallioniemi et al. (1992) developed a new fine scale molecular cytogenetic method called comparative genomic hybridization (CGH). This method allows for the detection of unbalanced chromosomal rearrangements (i.e., duplications, deletions, and copy number variation) between two sources of DNA. Originally the method was designed to detect chromosomal changes in tumor cells compared to normal cells (Kallioniemi et al., 1992). Later, it was used for sex chromosome detection using male and female DNA (e.g., Koubová et al., 2014; Pokorná et al., 2014). Finally, an interspecific design was developed to detect chromosomal rearrangements between species (Bi and Bogart, 2006; Symonová et al., 2015; de Oliveira et al., 2019). In most CGH studies done in birds, the chicken genome has been used as a reference with a microarray-based CGH platform (array-CGH) (Skinner et al., 2009, 2014; Völker et al., 2010). To our knowledge, no interspecific CGH comparisons have been performed to detect copy number variation between closely related bird species.

In this study, we compared the karyotypes of two closely related passerines species, the common nightingale (*Luscinia megarhynchos*) and the thrush nightingale (*Luscinia luscinia*), that diverged ∼1.8 Mya (Storchová et al., 2010) and currently hybridize in a secondary contact zone spanning Central and Eastern Europe (Reifová et al., 2011a). These species are separated by incomplete reproductive isolation, which is mainly caused by female-limited hybrid sterility (Reifová et al., 2011b; Mořkovský et al., 2018) and partial ecological divergence in sympatry (Reif et al., 2018; Sottas et al., 2018, 2020). In addition, divergence in sperm morphology might contribute to postmating prezygotic isolation (Albrecht et al., 2019). Of these species, the common nightingale’s karyotype has been previously described using classical cytogenetic analysis of the somatic metaphases (Bozhko, 1971). However, the karyotype of the thrush nightingale has yet to be determined.

Here we performed a cytogenetic analysis of the nightingale karyotypes to test whether changes in chromosomal structure might be linked to reproductive isolation between the species. To do so, we applied conventional and molecular cytogenetics methods to mitotic and meiotic spreads. These methods included C-banding, immunofluorescence staining of synapsed pachytene chromosomes, physical mapping of telomeric and 18S rDNA probes using FISH, and finally CGH.

## Materials and Methods

### Sampling Procedure

The sampling of the two nightingale species was carried out in allopatric regions (where only one of the two species occurs) to avoid possible sampling of interspecific hybrids. The common nightingale was sampled in South-western Poland by the Odra river, near the town Brzeg Dolny (N 51.2602°, E 16.7440°). The thrush nightingale was sampled in North-eastern Poland by the Narew river, near the town Łomża (N 53.1621°, E 22.1246°). In total, we sampled four common nightingales (one male, three females) and two thrush nightingales (one male, one female) for mitotic spreads and two males of each species for meiotic spreads. The birds were euthanized by a standard cervical dislocation and the tibia and testes were immediately dissected for the preparation of mitotic and meiotic chromosomal spreads, respectively. In addition, we collected a blood sample from the brachial vein from one female of each species. The blood sample was later used for DNA isolation and preparation of species-specific DNA probes for the interspecific comparative genomic hybridization (CGH) experiment. All individuals were sampled in May 2019, during the breeding season, and were captured using mist nets or collapsible traps. The work was approved by the General Directorate for Environmental Protection, Poland (permission no. DZP-WG.6401.03.123.2017.dl.3).

### Mitotic Chromosome Preparation and C-Banding

Bone marrow from the tibias of each bird was flushed out using a syringe needle with D-MEM medium (Sigma Aldrich) and cultivated in 5 ml of D-MEM medium (Sigma Aldrich) with 75 µl of colcemid solution (Roche) for 40 min at 37°C. After that, the cells were hypotonized in pre-warmed 0.075 M KCl solution for 25 min at 37°C. Finally, cells were washed four times with fixative solution (methanol:acetic acid, 3:1) and then stored at −20°C prior to use.

Chromosomal spreading was done using the air-drying technique followed by conventional Giemsa staining (5% Giemsa in 0.07 M phosphate buffer, pH 7.4). The C-banding method was applied for visualization of constitutive heterochromatin according to Sumner (1972). More specifically slides with chromosomal spreads were aged at 60°C for 1 h then successively soaked in 0.2 N HCl for 20 min at room temperature then in 5% Ba(OH)_2_ solution for 4–5 min at 45°C and subsequently in 2× SSC for 1 h at 60°C, with intermediate washes in distilled water. Finally, metaphases were mounted with 4′,6-diamidino-2-phenylindole (DAPI) in mounting medium Vectashield (Vector laboratories).

### Meiotic Chromosome Preparation and Immunostaining

Synaptonemal complex (SC) spreads were prepared from the testes of reproductively active males following Peters et al. (1997). Briefly, the left testis was cut into two pieces and placed in hypotonic solution (30 mM Tris, 50 mM sucrose, 17 mM trisodium citrate dehydrate and 5 mM EDTA; pH 8.2) for 50 min. The testis tissue was then disaggregated in 200 µl of 100 mM sucrose and the resulting cell suspension was applied in 40 µl drops and spread onto a slide previously treated with 1% PFA and 0.15% of Triton X100 (Sigma Aldrich). All slides were placed in a humid chamber for 90 min and washed for 2 min in 1× PBS. Slides were directly used for immunostaining.

Immunostaining was performed according to Moens et al. (1987) using the following primary antibodies: rabbit polyclonal anti-SYCP3 antibody (ab15093, Abcam) recognizing the lateral elements of the synaptonemal complex (dilution 1:200), and human anticentromere serum (CREST, 15-234, Antibodies Incorporated) binding kinetochores (dilution 1:50). The corresponding secondary antibodies were Alexa-594-conjugated goat anti-Rabbit IgG (H + L) (A32740, Invitrogen; dilution 1:200) and Alexa-488-conjugated goat anti-Human IgG (H + L) (A-11013, Invitrogen; dilution 1:200). Primary and secondary antibodies were diluted in PBT (3% BSA and 0.05% Tween 20 in 1× PBS) and incubated in a humid chamber for 90 min. Slides were washed three times in 1× PBS and dehydrated through an ethanol row (50, 70 and 96%, 3 min each). Finally, all slides were dried and stained with DAPI in mounting medium Vectashield (Vector laboratories).

### Fluorescence *In Situ* Hybridization (FISH) With Telomeric and 18S rRNA Probes

Telomeric repeat probe (TTAGGG)_*n*_ was applied to meiotic and mitotic spreads using FISH. In both experiments, the telomeric repeat sequences were detected using a commercial kit probe directly labelled with Cy3 (DAKO). We followed the manufacturer’s instructions, with the hybridization extended to 1.5 h.

The distribution of 18S rDNA genes was analyzed on mitotic spreads using FISH. The 18S rDNA probe was generated by PCR amplification and nick-translation labelling according to the protocol of Cioffi et al. (2009). The template genomic DNA originated from a reptile species, slow-worm (*Anguis fragilis*), and the PCR product was 1,456 bp in length (sequence is provided in Supplementary Table 1). The probe showed high sequence similarity with the rDNA of several bird species, *Gallus gallus* (97.73%), *Hirundo rustica* and *Motacilla alba* species (both 97.11%), and thus was considered similar enough to detect the distribution of 18S rDNA clusters in the nightingale species. Slides were aged for 1 h at 60°C, then treated with RNase for 1 h at 37°C and washed three times for 5 min in 2× SSC. Chromosomes were treated with pepsin for 3 min at 37°C and then fixed for 10 min in 1% formaldehyde solution. Slides were dehydrated in an ethanol row (70, 85 and 96%, 3 min each) and air-dried. The chromosomes were denatured in 75% formamide/2× SSC at 76°C for 3 min followed by dehydration in an ethanol row. Meanwhile, the probe was denatured at 80°C for 6 min and chilled on ice for 10 min prior to the hybridization. The probe-chromosome hybridization was performed overnight at 37°C. Post-hybridization washes were performed three times for 5 min in 50% formamide/2× SSC at 37°C and washed twice for 5 min in 2× SSC and finally for 5 min in 4× SSC/0.05% Tween 20 (Sigma Aldrich). Slides were first incubated for 30 min at 37°C with 4× SSC/5% blocking reagent (Roche), then the probe signal was detected by 4× SSC/5% blocking reagent mixed with fluorescein conjugated avidin (Vector laboratories) for 30 min at 37°C, followed by three washes in 4× SSC/0.05% Tween 20 for 5 min. Slides were then incubated with biotinylated anti-avidin (Vector laboratories) for 30 min at 37°C, followed by a second round of fluorescein conjugated avidin treatment for signal amplification. Slides were finally washed in 4× SSC/0.05% Tween 20 twice for 5 min, dehydrated through an ethanol row and air-dried. Chromosomes were stained with DAPI in mounting medium, Vectashield (Vector laboratories).

### Comparative Genomic Hybridization (CGH)

**FIGURE 1:**
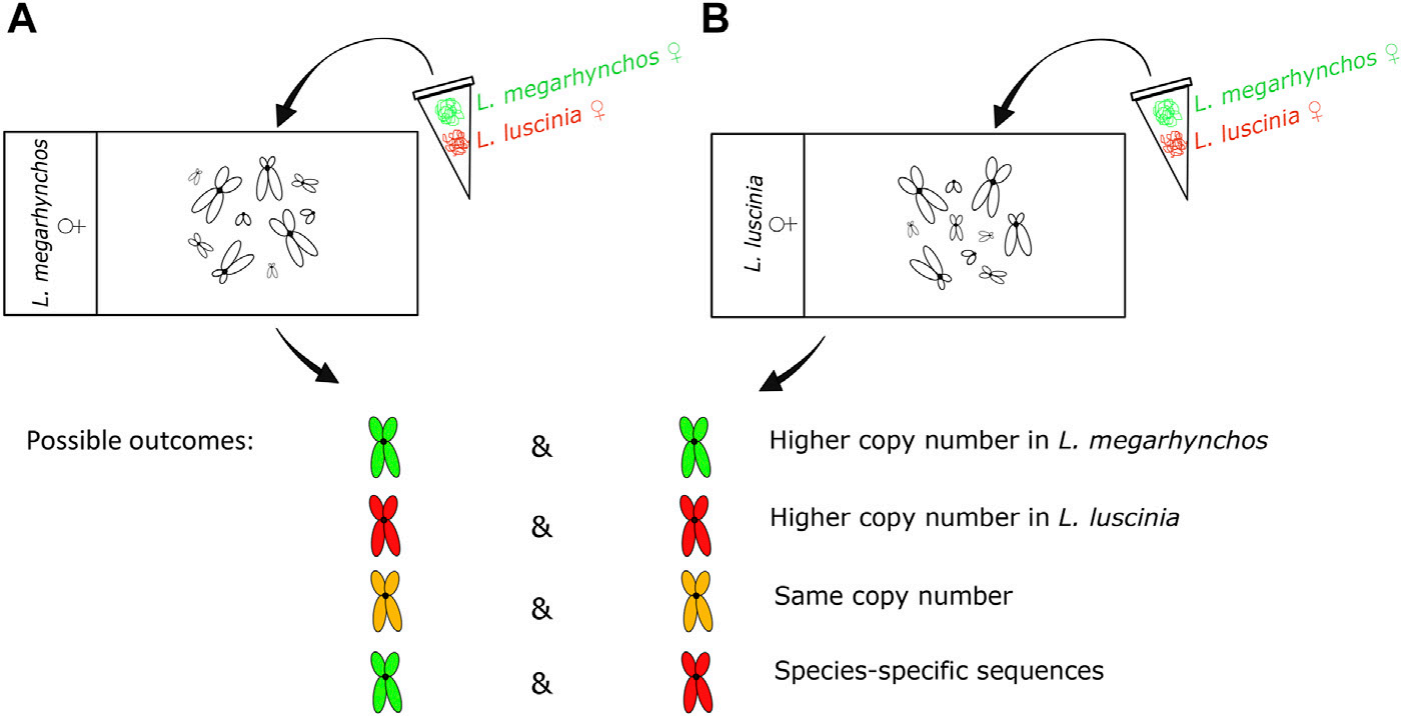
Design and possible outcomes of the interspecific comparative genomic hybridization (CGH) experiment. The genomic probe of the common nightingale (*L. megarhynchos*) (stained green) and the thrush nightingale (*L. luscinia*) (stained red) were hybridized on the metaphase of common nightingale **(A)** and thrush nightingale **(B)**. Whereas in the first three outcomes the same repetitive sequences exist in both species and the color reflects differences in their copy number, the last outcome points to the existence of species-specific repetitive sequences.

The CGH experiment was performed with (i) common nightingale and (ii) thrush nightingale metaphase chromosomes. In both cases, equal concentrations of DNA probes from the common nightingale and the thrush nightingale were hybridized to the chromosomes (Figure 1) following the procedure described in Symonová et al. (2015) with slight modification. The DNA probe was labelled by biotin (detected by streptoavidin-FITC, green) in the common nightingale and digoxigenin (detected by antidigoxigenine-rhodopsine, red) in the thrush nightingale. In both experimental designs, the green signal suggests a higher copy number of a particular repetitive sequence in the common nightingale, while the red signal indicates a higher copy number in the thrush nightingale. An intermediate yellow/orange signal suggests the same copy number in both species. Finally, a green signal in one design, while red in the other, indicates the presence of species-specific sequence (Figure 1).

Genomic DNA for the preparation of probes was extracted from blood samples using DNeasy Blood and Tissue Kit (Qiagen). The probes were prepared by nick translation (Abbott Laboratories) according to the manufacturer’s protocol and labelled with biotin-dUTP (Roche) and digoxigenin-dUTP (Roche). The nick translation took place at 15°C for 2 h. From each sample, 1 µg of DNA was co-precipitated overnight at −20°C with an additional 5 µl of salmon sperm DNA (10 mg/ml, Sigma Aldrich), 3 µl of 3 M sodium acetate (pH 5.2) and 2.5 volume of 96% ethanol. After precipitation, the dry pellets were resuspended in 11 µl of hybridization buffer for each slide (50% formamide in 2× SSC, 10% dextran sulfate, 10% sodium dodecyl sulfate and 1× Denhardt’s buffer, pH 7.0), denatured at 86°C for 6 min and then chilled on ice for 10 min prior to hybridization.

Metaphase slides were prepared by treatment with RNase and pepsin before being fixed with 1% formaldehyde, dehydrated through an ethanol row (70, 85 and 96%, 3 min each) and air-dried. Chromosomes were then denatured in 75% formamide/2× SSC at 76°C for 3 min and dehydrated again in an ice cold ethanol raw (70, 80 and 96%, 3 min each). Finally, 11 µl of the probe mix was applied to each slide and hybridization took place at 37°C for 48 h. The same probe mix was applied to metaphases of both nightingale species in the two CGH designs.

Post-hybridization washes were performed two and three times in 50% formamide/2× SSC and 1× SSC at 44°C, respectively. Each slide was incubated with 100 μl of 4× SSC/5% blocking reagent (Roche) at 37°C for 30 min and then with 100 μl of detection mix containing 4× SSC/5% blocking reagent, 2 μl of streptavidin-FITC (Vector Laboratories) and 1 μl of anti-digoxigenin-rhodamine (Roche) at 37°C for 1 h. The slides were subsequently washed in 4× SSC/0.01% Tween 20 (Sigma Aldrich) at 44°C, dehydrated through an ethanol row (70, 85 and 96%, 3 min each) and air-dried. Finally, the chromosomes were counterstained with DAPI in mounting medium Vectashield (Vector laboratories).

### Microscopy and Image Processing

Mitotic spreads were captured with an Axio Imager Z2 microscope (Zeiss) equipped with the automatic Metafer-MSearch scanning platform and a CoolCube 1 b/w digital camera (MetaSystems). Meiotic spreads were analyzed using an Olympus BX53 fluorescence microscope (Olympus) equipped with a DP30BW digital camera (Olympus). Ikaros karyotyping software (Metasystems) was used to remove the background from the metaphase images and to arrange the karyotypes. The colors of the C-banded metaphase images were inverted. All color images were captured in black and white, and later pseudocolored and superimposed using Adobe Photoshop software (version CC 2017).

A total of 18 and 17 metaphases from bone marrow were analyzed for the common nightingale and the thrush nightingale, respectively. The W chromosome was detected by C-banding due to its heterochromatic character. The Z chromosome was identified by comparing the female (ZW) and male (ZZ) metaphases. The size of each bivalent and its arm ratio was measured using the LEVAN plugin in the program ImageJ (Schindelin et al., 2012). Depending on the position of the centromere, we distinguished for each macrochromosome whether it was telocentric, acrocentric, submetacentric or metacentric chromosomes (Levan, 1964). For microchromosomes, the telocentric/acrocentric categories and submetacentric/metacentric categories were merged as they were difficult to distinguish clearly. The Z chromosome was identified among bivalents based on its relative size to the other macrochromosomes identified on the mitotic spreads. Chromosomes were measured in 15 pachytene cells in each species.

The metaphase chromosomes with applied CGH were analyzed using Photoshop (version CC 2017). For each CGH design, three cells were analyzed and compared. The centromeric red and green signals of the nine largest macrochromosomes and the sex chromosomes were measured using the histogram color tools. Each metaphase was measured three times to reduce the technical error associated with the signal measurement. The color ratio was calculated using the median value of both colors, after their normalization using the total red and green signal color.

## Results

### Mitotic and Meiotic Karyotypes

**FIGURE 2:**
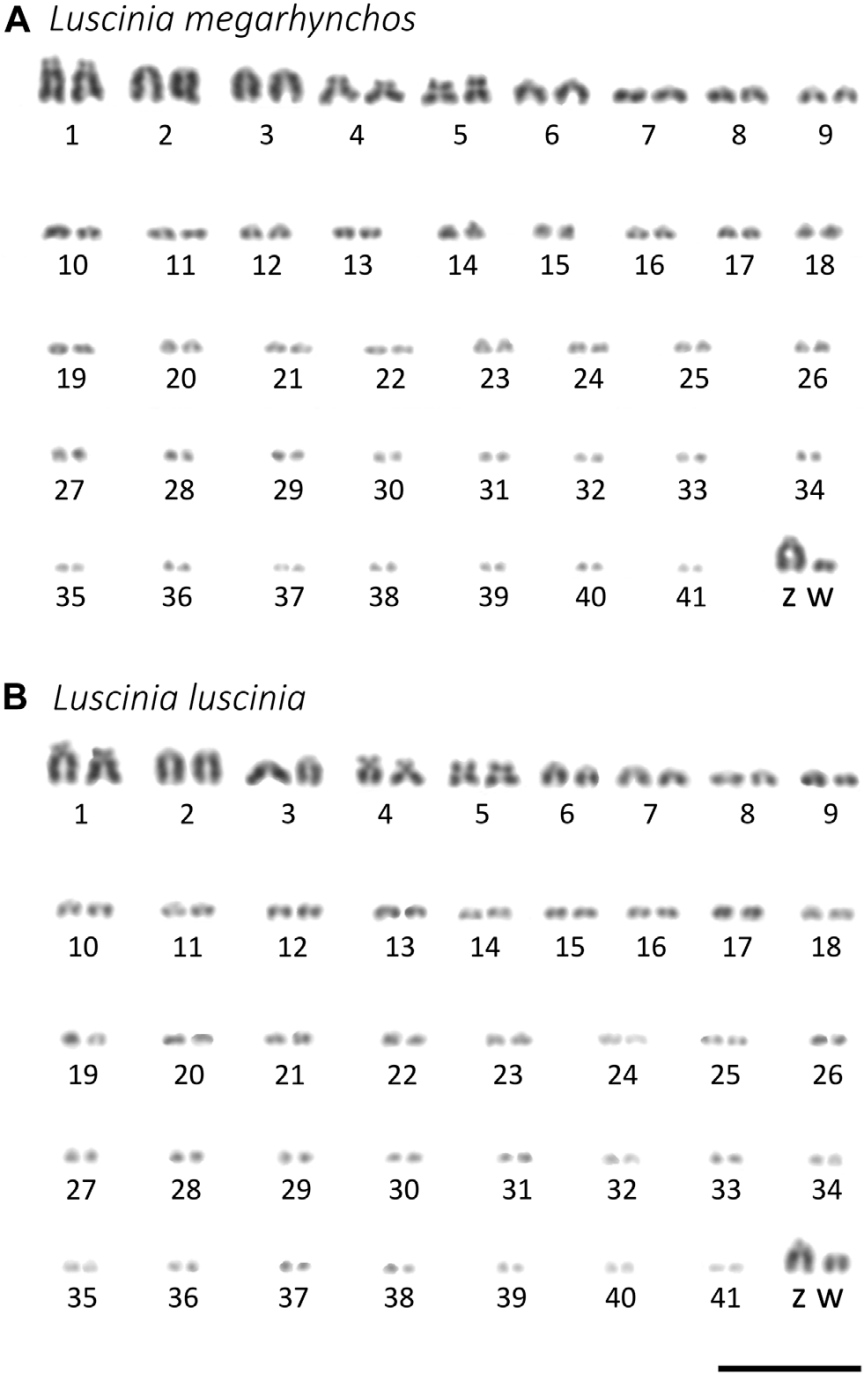
Karyotypes of the common nightingale (*L. megarhynchos*) (**A**) and the thrush nightingale (*L. luscinia*) (**B**) females arranged after Giemsa staining. W chromosome was detected using C-banding. Scale bar = 10 μm.

**FIGURE 3:**
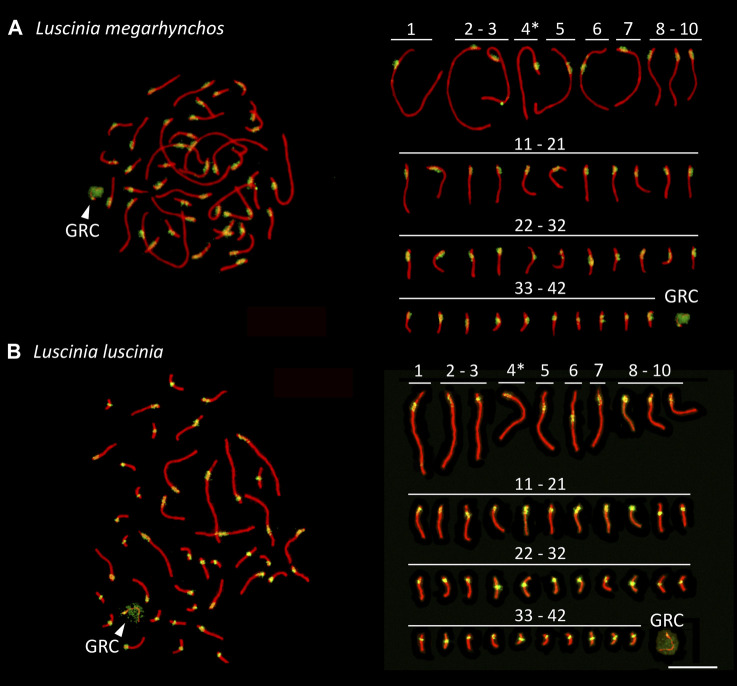
Synaptonemal complex spreads made from testes of the common nightingale (*L. megarhynchos*) **(A)** and the thrush nightingale (*L. luscinia*) **(B)**, immunostained with antibodies against the lateral elements of the synaptonemal complex, SYCP3 (red) and against centromere proteins (green). The presumed Z chromosome bivalents are indicated with an asterisk and the germline restricted chromosome (GRC) with an arrowhead. Scale bar = 10 µm.

Both species showed a more or less continuous decrease in chromosome size without a clear distinction between macrochromosomes and microchromosomes (Figure 2). We categorized the 10 largest chromosome pairs including the sex chromosomes to be macrochromosomes with the remaining chromosomes considered to be microchromosomes. Because the mitotic chromosomes were not always well spread, it was difficult to estimate the diploid chromosome number from metaphase spreads. This was especially true with respect to the number of microchromosomes. We thus calculated the diploid chromosome number for both species based on immunostained meiotic spreads (Hale et al., 1988; del Priore and Pigozzi, 2020). Both the common nightingale and the thrush nightingale consistently displayed 42 bivalents, establishing a diploid chromosome number of 84 for each species (Figure 3). In addition to these bivalents, both species displayed an extra univalent chromosome in male germ cells, corresponding to the GRC (Figure 3). The GRC was stained weaker by anti-SYCP3 antibody and showed a CREST signal not only in the centromere, but along the whole chromosome, as has been described previously in other passerine species (Torgasheva et al., 2019).

Staining of centromeres in meiotic chromosomes by the CREST antibody allowed us to estimate the arm ratio for each chromosome and compare chromosome morphology between species in a more precise way than was possible with mitotic chromosomes. The ten largest chromosomes had the same morphology between the species, suggesting that no chromosomal rearrangements that would have changed the position of the centromere occurred on these chromosomes. In both species, the largest chromosome, SC1, was identified as acrocentric, SC2 to SC4 as telocentric, SC5 as submetacentric, SC6 as metacentric and SC7 to SC9 as telocentric. However, SC10 was telocentric in the common nightingale, but acrocentric in the thrush nightingale (Figure 3; Supplementary Table 2), indicating that some rearrangements might have occurred on this chromosome.

**FIGURE 4:**
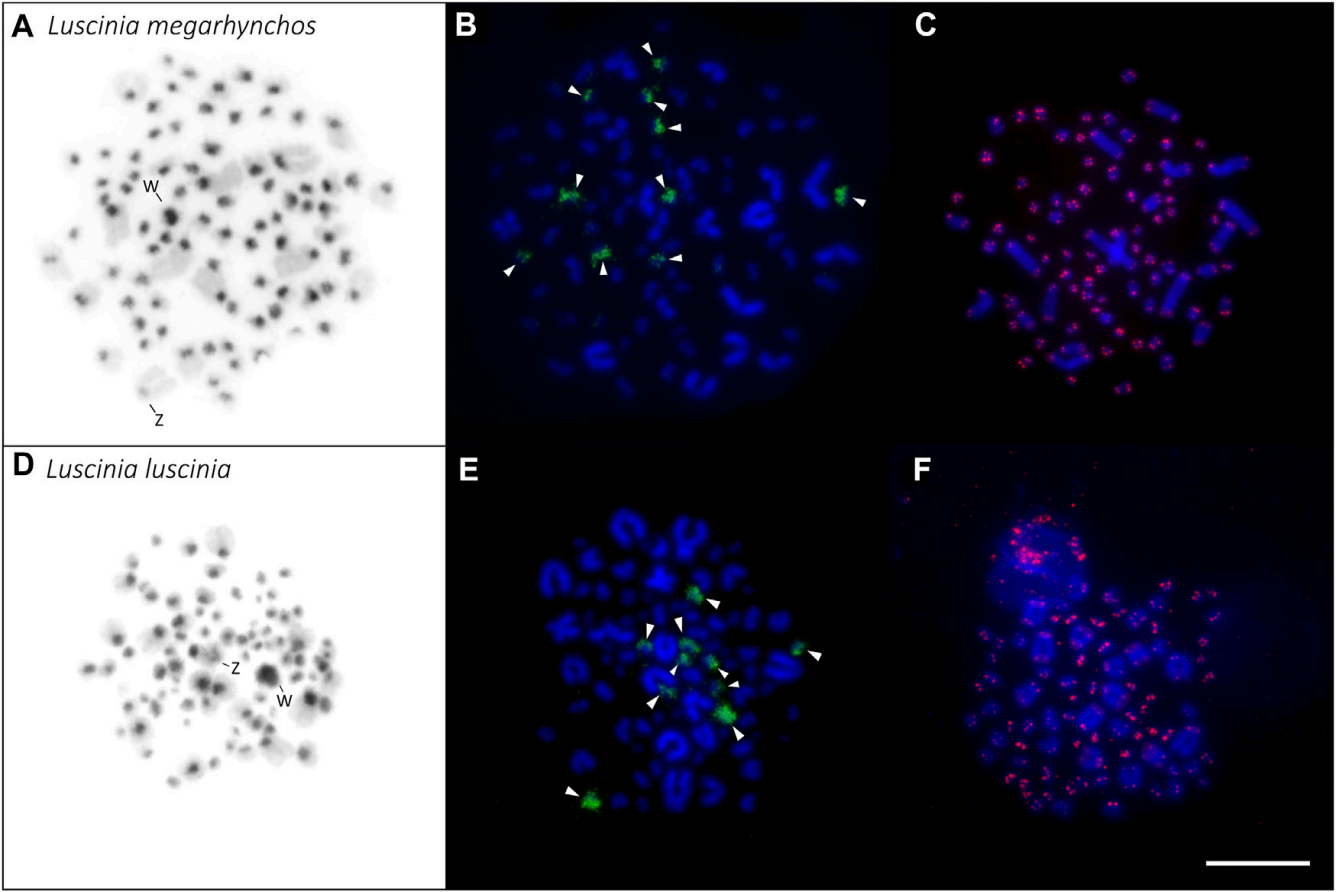
Distribution of heterochromatin **(A,D)**, 18S rDNA clusters **(B,E)** and telomeric repeats **(C,F)** in the karyotype of the two nightingale species. C-banding in the common nightingale (*L. megarhynchos*) **(A)** and the thrush nightingale (*L. luscinia*) **(D)** female karyotypes. Sex chromosomes are indicated in both karyotypes. rDNA clusters (green) in the common nightingale **(B)** and the thrush nightingale **(E)**. Arrowheads point to 10 microchromosomes displaying a rDNA signal. Telomeric repeat sequences (TTAGGG)_*n*_ (red) in the common nightingale **(C)** and the thrush nightingale **(F)**. Scale bar = 10 µm.

Based on the comparison of male and female mitotic spreads, we identified the Z chromosome as the fourth largest chromosome in both species and in both species it was telocentric. The W chromosome was also telocentric, with a size between the 10th and 11th chromosome in the common nightingale and between the nineth and 10th chromosome in the thrush nightingale (Figure 4).

The morphology of the microchromosomes slightly differed between the two species with 17 acrocentric/telocentric and 15 submetacentric/metacentric microchromosomes in the common nightingale and 19 acrocentric/telocentric and 13 submetacentric/metacentric microchromosomes in the thrush nightingale (Figure 3; Supplementary Table 2). The GRC was present in both nightingale species, with its size corresponding to a microchromosome.

### Distribution of Heterochromatin, 18S rDNA Genes and Telomeric Repeats in the Two Species

The distribution of the constitutive heterochromatin revealed by C-banding displayed the same pattern in the two nightingale species. C-banding signals mainly occurred in the centromeric regions of macrochromosomes and microchromosomes, but sometimes the signal covered the entire microchromosome. The W chromosome displayed a large C-banding signal in both species, but the signal was slightly larger in the thrush nightingale than in the common nightingale (Figure 4). In both species, the Z chromosome had a small heterochromatic band in the centromeric region (Figure 4).

18S rDNA clusters were consistently located on 10 microchomosomes in both species (Figure 4). The same number and distribution of the 18S rDNA clusters suggests that no rearrangements that would include rDNA genes had occurred between the two species.

The telomeric motif (TTAGGG)_*n*_ was detected at the terminal regions of all chromosomes. No interstitial telomeric signal was detected (Figure 4; Supplementary Figure 1). This can be seen in both the mitotic (Figure 4) and meiotic spreads (Supplementary Figure 1).

### Divergence of Centromeric Repeats Between the Two Nightingale Species

**FIGURE 5:**
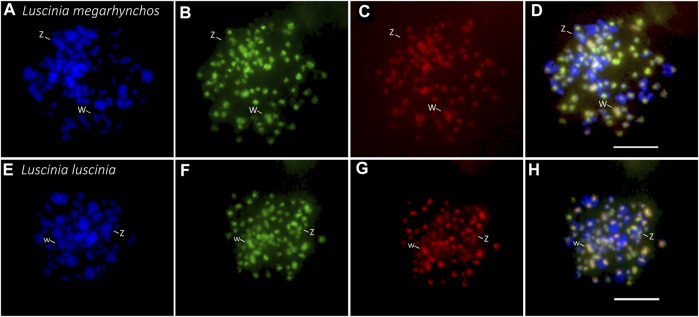
Interspecific comparative genomic hybridization (CGH) in two nightingale species. Female probes of the common nightingale (*L. megarhynchos*) and the thrush nightingale (*L. luscinia*) were labelled by streptavidin-FITC (green) and anti-digoxigenin-rhodamine (red), respectively, and hybridized on common nightingale (**A–D**), and thrush nightingale (**E–H**) metaphase spreads. The first column displays DAPI images (blue) (**A,E**); the second column displays metaphases with the common nightingale DNA probe signal (green) (**B,F**); the third column displays metaphases with the thrush nightingale DNA probe signal (red) (**C,G**); the fourth column displays the merged colors of both genomic DNA probes and DAPI staining (**D,H**). Scale bar = 10 μm.

In both interspecific CGH experimental designs (i.e., with the common nightingale and the thrush nightingale metaphases, Figure 1), the distribution pattern of the probe signal was similar to that of the heterochromatin from the C-banding experiment, meaning that the probe signal was brightest in the centromeric regions of the macrochromosomes and microchromosomes (Figures 4, 5). In some microchromosomes, the whole chromosome appeared to be generating signal, however, due to the small size of chromosomes and the signal strength of the probes, this might still only represent centromeric binding.

**FIGURE 6:**
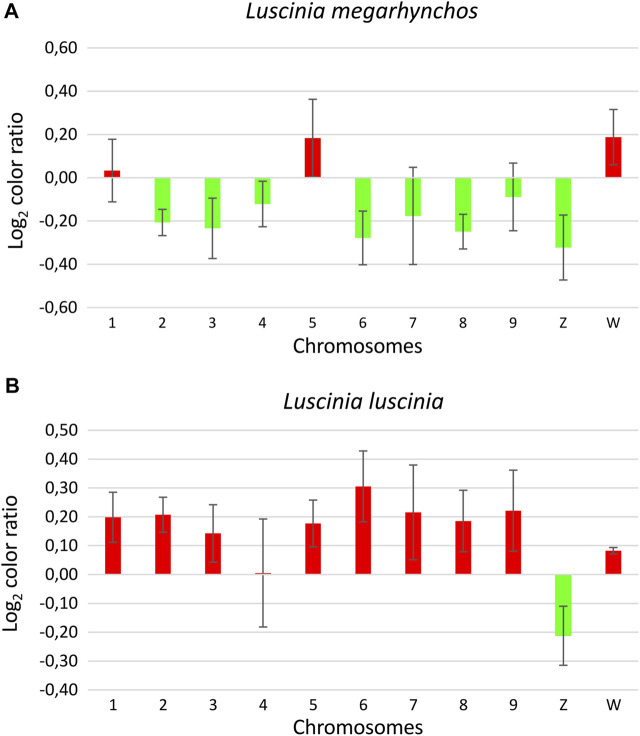
Ratio of the green and red signal intensity at centromeric regions from the interspecific comparative genomic hybridization (CGH) experiment with the common nightingale (*L. megarhynchos*) metaphases **(A)** and the thrush nightingale (*L. luscinia*) metaphases **(B)**. Log_2_ color ratio is shown for nine macrochromosomes and the sex chromosomes. Values lower than zero represent higher signal of common nightingale probes (green) and values higher than zero represent higher signal of thrush nightingale probes (red). Bar charts are based on the Log_2_ ratio with error bars representing the standard error.

Interestingly, the centromeric regions of the nine largest autosomes were mostly green (common nightingale probe) in the CGH with common nightingale metaphases and red (thrush nightingale probe) in the CGH with thrush nightingale metaphases, suggesting sequence divergence of repetitive elements in the centromeric regions. The exceptions were the first and fifth chromosome pairs, which showed an increased red signal in both CGH designs, indicating a higher copy number of centromeric repetitive elements in the thrush nightingale genome. The fourth pair produced variable signals across the three metaphases, making the results difficult to interpret (Figures 5, 6; Supplementary Table 3).

The whole W chromosome displayed a higher red signal in both interspecific CGH designs, indicating a higher number of repetitive elements in the thrush nightingale genome. Contrastingly, the probe signal in the centromeric region of the Z chromosome was greener in both the common nightingale and the thrush nightingale experimental designs (Figures 5, 6; Supplementary Table 3). Thus, the Z chromosome seems to have a higher copy number of centromeric repetitive elements in the common nightingale compared to the thrush nightingale.

The centromeric regions of microchromosomes were mostly green in the CGH with the common nightingale metaphases and red in the CGH with the thrush nightingale metaphases, suggesting sequence divergence of centromeric repeats on most microchromosomes (Figure 5).

## Discussion

In this study, we compared the chromosomal structure in two closely related passerine species, the common nightingale and the thrush nightingale, that show partial reproductive isolation caused mainly by hybrid female sterility and ecological differentiation (Storchová et al., 2010; Reifová et al., 2011b; Mořkovský et al., 2018; Reif et al., 2018; Sottas et al., 2018). We found that the two species have the same diploid chromosome number 2n = 84 and both possess a micro GRC in the germ cells. However, a few subtle changes in chromosome morphology imply that some chromosomal rearrangements might have occurred between the species. Interestingly, the interspecific CGH experiment suggests that the two nightingale species might have diverged in centromeric repetitive sequences on most chromosomes. Some chromosomes showed changes in copy number of centromeric repeats between the species.

Changes in chromosomal structure are assumed to play an important role in the origin of reproductive isolation. They can for example impair meiosis in hybrids leading to hybrid sterility, or suppress recombination linking together species-specific combinations of alleles, which may help to maintain species differentiation in the face of gene flow (Rieseberg, 2001; Ortíz-Barrientos et al., 2002; Butlin, 2005). The two nightingale species have very similar karyotypes, with 10 macrochromosomes (including the sex chromosomes) and 32 microchromosomes. This observed diploid chromosome number is consistent with the previously described chromosome number for the common nightingale (Bozhko, 1971). The diploid chromosome number in other species of the family Muscicapidae varies between 2n = 64 and 2n = 86 (Udagawa, 1955; Bulatova and Panov, 1973; Mittal and Satija, 1978; Bulatova, 1981; Degrandi et al., 2020a). Thus, although some large-scale chromosomal rearrangements occurred between more distantly related species of the Muscicapidae family, the closely related nightingale species seem to have the same chromosome number, which is consistent with the generally slow evolution of bird karyotypes (Christidis, 1990; Pichugin et al., 2001; Masabanda et al., 2004; Griffin et al., 2007; Ellegren, 2010; Nanda et al., 2011).

The distribution of constitutive heterochromatin blocks observed in the nightingale species is typical for passerine birds (e.g., Kretschmer et al., 2014; Barcellos et al., 2019). The larger heterochromatin block on the W in the thrush nightingale suggests that this species might have accumulated more repetitive sequences on this chromosome compared to the common nightingale. This was supported by the CGH experiment demonstrating that the W of the thrush nightingale show a higher copy number of repetitive sequences than the common nightingale. Together these results suggest the relatively fast evolution of the W repetitive content, which might theoretically contribute to reproductive isolation between the species (Peona et al., 2021).

The rDNA clusters are considered as hotspots of chromosomal breakage due to their repetitive nature as well as their intense transcriptome activity (Huang et al., 2008; Cazaux et al., 2011). In birds, a large variation in the number of chromosome pairs bearing the 18S rDNA cluster is observed, ranging from one to six or seven pairs, with the majority of species displaying only one chromosome pair with rDNA cluster (Degrandi et al., 2020b). Both nightingale species showed rDNA FISH signal on five microchromosome pairs, suggesting that no rDNA associated chromosomal changes have occurred between these two species. Interestingly, five chromosome pairs bearing rDNA clusters is the highest number found in passerines so far (Degrandi et al., 2020b). In the closest related species, where rDNA has been cytogenetically localized, *Turdus rufiventris* and *Turdus albicolis*, belonging to the Turdidae family, only three and two microchromosome pairs, respectively, bear rDNA (Kretschmer et al., 2014). Such differences in the number of rDNA clusters might result from chromosome translocations, transpositions and duplications mediated by transposable elements or ectopic recombination (Nguyen et al., 2010; Teixeira et al., 2021).

Telomeric tandem repeats (TTAGGG)_*n*_ are normally found at the end of chromosomes but can sometimes be present also inside the chromosomes. Such interstitial telomere sites (ITSs) may result from chromosome translocation or fusions (Nanda and Schmid, 1994; Nanda et al., 2002), although not all chromosome fusions lead to ITSs (de Oliveira et al., 2005; Nishida et al., 2008). In birds, a high number of ITSs have been found in Ratites and Galliformes (Nanda and Schmid, 1994; Nanda et al., 2002), however, in passerines only a few or no ITSs have been identified (Nanda et al., 2002; Derjusheva et al., 2004). No ITSs were detected in either of the nightingale species, providing more evidence for a conserved chromosomal structure in the two species.

It has been shown that the GRC, an extra chromosome occurring in the germline of songbirds (Pigozzi and Solari, 1998, 2005), is highly variable in its size among species (Torgasheva et al., 2019; Malinovskaya et al., 2020). Torgasheva et al. (2019) compared the size of this chromosome in 16 passerine species belonging to nine families and showed that in 10 of them the GRC is a big macrochromosome, while in six it is a small microchromosome. Our results showed that both nightingale species had a small GRC, comparable in size to a microchromosome.

Despite the same chromosome number in the two nightingale species, we observed a few small changes in the centromere position on one macrochromosome and several microchromosomes, suggesting that some intrachromosomal rearrangements might have occurred between these two species. More detailed analyses of nightingale karyotypes and their genomic sequence will be needed, however, to confirm the existence of structural variants between the two species and determine their size and content. We should also note that our cytogenetic approach cannot detect smaller chromosomal rearrangements, which do not change the position of the centromere, result in ITSs or change the number of rDNA clusters. Analysis of high-quality chromosome-level genome assemblies of the two species could shed more light on the possible smaller-scale structural changes between the species.

Another interesting difference in the chromosome structure of the two nightingale species was revealed by the interspecific CGH experiment. This experiment suggested that some chromosomes have different copy numbers of centromeric repeats between the two species. In addition, many macrochromosomes and microchromosomes displayed higher conspecific signals in the CGH experiment, suggesting that the two species have diverged in their centromeric repeat sequences. Our observation is consistent with other studies in birds (Ellegren et al., 2012), as well as other taxa (Haaf and Willard, 1997; Bensasson et al., 2008; Pertile et al., 2009; de Sassi et al., 2021; Oliveira et al., 2021), showing fast evolution of centromeric sequences. For example, comparison of the whole genome sequences of two closely related species of *Ficedula* flycatchers, which also belong to the Muscicapidae family, revealed that the centromeres were among the most differentiated regions of the genome between the species (Ellegren et al., 2012).

The rapid divergence of the centromeric sequences or their copy number, between species is assumed to be the result of centromere-associated female meiotic drive, where some centromeric sequences can bias their transmission to the egg, leaving others to end up in the polar bodies (Henikoff, 2001; Pardo-Manuel de Villena and Sapienza, 2001). This can lead to a swift fixation of particular centromeric sequences in the population and a fast divergence of centromeric repeats, or their copy number, between the species. However, distorting the transmission ratio can sometimes be harmful to the organism, for example, if it is linked to the sex chromosomes and leads to a sex ratio distortion. In such cases, it is often associated with the evolution of drive suppressors (McLaughlin and Malik, 2017). Interestingly, while most large autosomes showed species-specific sequences in our CGH experiment, sex chromosomes showed differences in the copy number of centromeric repeats, but not species-specific sequences. This suggests that the evolution of centromeric repeats on the sex chromosomes might be constrained by the sex ratio effect of sex chromosome linked meiotic drive.

It has been demonstrated that divergence of centromeric sequences between species may lead to female meiotic drive in interspecific hybrids (Chmátal et al., 2014; Akera et al., 2019; Knief et al., 2020). Theoretically, the divergence of centromeres could, in an extreme case, also cause the sterility of female hybrids and thus contribute to reproductive isolation between species (Hurst and Pomiankowski, 1991; Phadnis and Orr, 2009; Zhang et al., 2015). In nightingales, consistent with Haldane’s rule (Haldane, 1922), F_1_ hybrid females are sterile, while F_1_ males are fertile (Reifová et al., 2011b; Mořkovský et al., 2018). It is thus possible that divergence in centromeric sequences between the two nightingale species could contribute to female-limited hybrid sterility. Further studies of centromere composition in the two nightingale species should be done to explore this possibility.

In conclusion, although the two nightingale species have very similar karyotypes, it is possible that a small number of chromosomal rearrangements have occurred between them and may contribute to reproductive isolation between the species. Interestingly, the two species appear to differ in their centromeric sequences. Such divergence could cause female meiotic drive or female sterility in interspecific hybrids. Further studies are, however, needed to confirm the presence of structural variants and diverged centromeric repeats in the two nightingale species and to examine their potential role in the nightingales’ speciation.

## Data Availability

The original contributions presented in the study are included in the article/Supplementary Material, further inquiries can be directed to the corresponding authors.

## References

[B1] Akera T. Trimm E. Lampson M. A. (2019). Molecular Strategies of Meiotic Cheating by Selfish Centromeres. Cell 178, 1132–1144. 10.1016/j.cell.2019.07.001 31402175 PMC6731994

[B2] Albrecht T. Opletalová K. Reif J. Janoušek V. Janoušek L. Cramer E. R. A. (2019). Sperm Divergence in a Passerine Contact Zone: Indication of Reinforcement at the Gametic Level. Evolution 73, 202–213. 10.1111/evo.13677 30597549

[B3] Aslam M. L. Bastiaansen J. W. Crooijmans R. P. Vereijken A. Megens H.-J. Groenen M. A. (2010). A SNP Based Linkage Map of the turkey Genome Reveals Multiple Intrachromosomal Rearrangements between the Turkey and Chicken Genomes. BMC Genomics 11, 647. 10.1186/1471-2164-11-647 21092123 PMC3091770

[B4] Auer H. Mayr B. Lambrou M. Schleger W. (1987). An Extended Chicken Karyotype, Including the NOR Chromosome. Cytogenet. Cel Genet. 45, 218–221. 10.1159/000132457 3691189

[B5] Axelsson E. Webster M. T. Smith N. G. C. Burt D. W. Ellegren H. (2005). Comparison of the Chicken and turkey Genomes Reveals a Higher Rate of Nucleotide Divergence on Microchromosomes Than Macrochromosomes. Genome Res. 15, 120–125. 10.1101/gr.3021305 15590944 PMC540272

[B6] Barcellos S. A. Kretschmer R. de Souza M. S. Costa A. L. Degrandi T. M. dos Santos M. S. (2019). Karyotype Evolution and Distinct Evolutionary History of the W Chromosomes in Swallows (Aves, Passeriformes). Cytogenet. Genome Res. 158, 98–105. 10.1159/000500621 31158838

[B7] Bensasson D. Zarowiecki M. Burt A. Koufopanou V. (2008). Rapid Evolution of Yeast Centromeres in the Absence of Drive. Genetics 178, 2161–2167. 10.1534/genetics.107.083980 18430941 PMC2323805

[B8] Bi K. Bogart J. P. (2006). Identification of Intergenomic Recombinations in Unisexual Salamanders of the Genus *Ambystoma* by Genomic *In Situ* Hybridization (GISH). Cytogenet. Genome Res. 112, 307–312. 10.1159/000089885 16484787

[B9] Bozhko S. I. (1971). Karyotypes of Two Bird Species, Nightingale (*Luscinia megarhynchos* Brehm) and Song Trush (*Turdus philomelos* Brehm). Acta Biologica Debrecina 9, 131–136.

[B10] Bruders R. Van Hollebeke H. Osborne E. J. Kronenberg Z. Maclary E. Yandell M. (2020). A Copy Number Variant Is Associated with a Spectrum of Pigmentation Patterns in the Rock Pigeon (*Columba livia*). Plos Genet. 16, e1008274. 10.1371/journal.pgen.1008274 32433666 PMC7239393

[B11] Bulatova N. S. (1981). A Comparative Karyological Study of Passerine Birds, 15. Praha: Academia Nakladatelstvi Československé akademie věd, 1–44.

[B12] Bulatova N. S. Panov E. N. (1973). Comparative Analysis of Karyotypes of 18 Species Family Turdidae (Aves). Caryologia 26, 229–244. 10.1080/00087114.1973.10796539

[B13] Burt D. W. (2002). Origin and Evolution of Avian Microchromosomes. Cytogenet. Genome Res. 96, 97–112. 10.1159/000063018 12438785

[B14] Butlin R. K. (2005). Recombination and Speciation. Mol. Ecol. 14, 2621–2635. 10.1111/j.1365-294X.2005.02617.x 16029465

[B15] Cazaux B. Catalan J. Veyrunes F. Douzery E. J. Britton-Davidian J. (2011). Are Ribosomal DNA Clusters Rearrangement Hotspots? A Case Study in the Genus *Mus* (Rodentia, Muridae). BMC Evol. Biol. 11, 124. 10.1186/1471-2148-11-124 21569527 PMC3112088

[B16] Chmátal L. Gabriel S. I. Mitsainas G. P. Martínez-Vargas J. Ventura J. Searle J. B. (2014). Centromere Strength Provides the Cell Biological Basis for Meiotic Drive and Karyotype Evolution in Mice. Curr. Biol. 24, 2295–2300. 10.1016/j.cub.2014.08.017 25242031 PMC4189972

[B17] Christidis L. (1990). Animal Cytogenetics/Vol. 4, Chordata. 3. B, Aves/by Les Christidis. Editor John B. (Berlin: Gebrüder Bornträger).

[B18] Cioffi M. B. Martins C. Centofante L. Jacobina U. Bertollo L. a. C. (2009). Chromosomal Variability Among Allopatric Populations of Erythrinidae Fish *Hoplias malabaricus*: Mapping of Three Classes of Repetitive DNAs. Cytogenet. Genome Res. 125, 132–141. 10.1159/000227838 19729917

[B19] de M. C. Sassi F. Perez M. F. Oliveira V. C. S. Deon G. A. de Souza F. H. S. Ferreira P. H. N. (2021). High Genetic Diversity Despite Conserved Karyotype Organization in the Giant Trahiras from Genus *Hoplias* (Characiformes, Erythrinidae). Genes 12, 252. 10.3390/genes12020252 33578790 PMC7916553

[B20] de Oliveira E. A. Bertollo L. A. C. Rab P. Ezaz T. Yano C. F. Hatanaka T. (2019). Cytogenetics, Genomics and Biodiversity of the South American and African Arapaimidae Fish Family (Teleostei, Osteoglossiformes). PLoS ONE 14, e0214225. 10.1371/journal.pone.0214225 30908514 PMC6433368

[B21] de Oliveira E. H. C. Habermann F. A. Lacerda O. Sbalqueiro I. J. Wienberg J. Müller S. (2005). Chromosome Reshuffling in Birds of Prey: the Karyotype of the World's Largest eagle (Harpy eagle, *Harpia harpyja*) Compared to that of the Chicken (*Gallus gallus*). Chromosoma 114, 338–343. 10.1007/s00412-005-0009-5 16163545

[B22] Degrandi T. M. Barcellos S. A. Costa A. L. Garnero A. D. V. Hass I. Gunski R. J. (2020a). Introducing the Bird Chromosome Database: An Overview of Cytogenetic Studies in Birds. Cytogenet. Genome Res. 160, 199–205. 10.1159/000507768 32369809

[B23] Degrandi T. M. Gunski R. J. Garnero A. d. V. Oliveira E. H. C. d. Kretschmer R. Souza M. S. d. (2020b). The Distribution of 45S rDNA Sites in Bird Chromosomes Suggests Multiple Evolutionary Histories. Genet. Mol. Biol. 43, e20180331. 10.1590/1678-4685-GMB-2018-0331 32251493 PMC7197993

[B24] del Priore L. Pigozzi M. I. (2020). MLH1 Focus Mapping in the guinea Fowl (*Numida meleagris*) Give Insights into the Crossover Landscapes in Birds. PLoS ONE 15, e0240245. 10.1371/journal.pone.0240245 33017431 PMC7535058

[B25] Derjusheva S. Kurganova A. Habermann F. Gaginskaya E. (2004). High Chromosome Conservation Detected by Comparative Chromosome Painting in Chicken, Pigeon and Passerine Birds. Chromosome Res. 12, 715–723. 10.1023/B:CHRO.0000045779.50641.00 15505406

[B26] dos Santos M. d. S. Kretschmer R. Silva F. A. O. Ledesma M. A. O’Brien P. C. M. Ferguson-Smith M. A. (2015). Intrachromosomal Rearrangements in Two Representatives of the Genus *Saltator* (Thraupidae, Passeriformes) and the Occurrence of Heteromorphic Z Chromosomes. Genetica 143, 535–543. 10.1007/s10709-015-9851-4 26092368

[B27] Ellegren H. (2010). Evolutionary Stasis: the Stable Chromosomes of Birds. Trends Ecol. Evol. 25, 283–291. 10.1016/j.tree.2009.12.004 20363047

[B28] Ellegren H. Smeds L. Burri R. Olason P. I. Backström N. Kawakami T. (2012). The Genomic Landscape of Species Divergence in *Ficedula* Flycatchers. Nature 491, 756–760. 10.1038/nature11584 23103876

[B29] Ellegren H. (2013). The Evolutionary Genomics of Birds. Annu. Rev. Ecol. Evol. Syst. 44, 239–259. 10.1146/annurev-ecolsys-110411-160327

[B30] Feng S. Stiller J. Deng Y. Armstrong J. Fang Q. Reeve A. H. (2020). Dense Sampling of Bird Diversity Increases Power of Comparative Genomics. Nature 587, 252–257. 10.1038/s41586-020-2873-9 33177665 PMC7759463

[B31] Griffin D. K. Haberman F. Masabanda J. O’Brien P. Bagga M. Sazanov A. (1999). Micro- and Macrochromosome Paints Generated by Flow Cytometry and Microdissection: Tools for Mapping the Chicken Genome. Cytogenet. Genome Res. 87, 278–281. 10.1159/000015449 10702695

[B32] Griffin D. K. Robertson L. B. W. Tempest H. G. Skinner B. M. (2007). The Evolution of the Avian Genome as Revealed by Comparative Molecular Cytogenetics. Cytogenet. Genome Res. 117, 64–77. 10.1159/000103166 17675846

[B33] Haaf T. Willard H. F. (1997). Chromosome-specific α-satellite DNA from the Centromere of Chimpanzee Chromosome 4. Chromosoma 106, 226–232. 10.1007/s004120050243 9254724

[B34] Haldane J. B. S. (1922). Sex Ratio and Unisexual Sterility in Hybrid Animals. Journ. Gen. 12, 101–109. 10.1007/BF02983075

[B35] Hale D. W. Ryder E. J. Sudman P. D. Greenbaum I. F. (1988). Application of Synaptonemal Complex Techniques for Determination of Diploid Number and Chromosomal Morphology in Birds. The Auk 4, 776–779.

[B36] Henikoff S. Ahmad K. Malik H. S. (2001). The Centromere Paradox: Stable Inheritance with Rapidly Evolving DNA. Science 293, 1098–1102. 10.1126/science.1062939 11498581

[B37] Hoffmann A. A. Rieseberg L. H. (2008). Revisiting the Impact of Inversions in Evolution: From Population Genetic Markers to Drivers of Adaptive Shifts and Speciation? Annu. Rev. Ecol. Evol. Syst. 39, 21–42. 10.1146/annurev.ecolsys.39.110707.173532 20419035 PMC2858385

[B38] Homolka D. Ivanek R. Capkova J. Jansa P. Forejt J. (2007). Chromosomal Rearrangement Interferes with Meiotic X Chromosome Inactivation. Genome Res. 17, 1431–1437. 10.1101/gr.6520107 17717048 PMC1987340

[B39] Hooper D. M. Griffith S. C. Price T. D. (2019). Sex Chromosome Inversions Enforce Reproductive Isolation across an Avian Hybrid Zone. Mol. Ecol. 28, 1246–1262. 10.1111/mec.14874 30230092

[B40] Hooper D. M. Price T. D. (2017). Chromosomal Inversion Differences Correlate with Range Overlap in Passerine Birds. Nat. Ecol. Evol. 1, 1526–1534. 10.1038/s41559-017-0284-6 29185507

[B41] Hooper D. M. Price T. D. (2015). Rates of Karyotypic Evolution in Estrildid Finches Differ between Island and Continental Clades. Evolution 69, 890–903. 10.1111/evo.12633 25756186

[B42] Huang J. Ma L. Yang F. Fei S.-z. Li L. (2008). 45S rDNA Regions Are Chromosome Fragile Sites Expressed as Gaps *In Vitro* on Metaphase Chromosomes of Root-Tip Meristematic Cells in *Lolium* Spp. PLOS ONE 3, e2167. 10.1371/journal.pone.0002167 18478113 PMC2366065

[B43] Hurst L. D. Pomiankowski A. (1991). Causes of Sex Ratio Bias May Account for Unisexual Sterility in Hybrids: a New Explanation of Haldane's Rule and Related Phenomena. Genetics 128, 841–858. 10.1093/genetics/128.4.841 1916248 PMC1204557

[B44] Iskow R. C. Gokcumen O. Abyzov A. Malukiewicz J. Zhu Q. Sukumar A. T. (2012). Regulatory Element Copy Number Differences Shape Primate Expression Profiles. Proc. Natl. Acad. Sci. 109, 12656–12661. 10.1073/pnas.1205199109 22797897 PMC3411951

[B45] Jarvis E. D. Mirarab S. Aberer A. J. Li B. Houde P. Li C. (2014). Whole-genome Analyses Resolve Early Branches in the Tree of Life of Modern Birds. Science 346, 1320–1331. 10.1126/science.1253451 25504713 PMC4405904

[B46] Kallioniemi A. Kallioniemi O.-P. Sudar D. Rutovitz D. Gray J. W. Waldman F. (1992). Comparative Genomic Hybridization for Molecular Cytogenetic Analysis of Solid Tumors. Science 258, 818–821. 10.1126/science.1359641 1359641

[B47] King M. (1993). Species Evolution: The Role of Chromosome Change. Cambridge: Cambridge University Press.

[B48] Kinsella C. M. Ruiz-Ruano F. J. Dion-Côté A.-M. Charles A. J. Gossmann T. I. Cabrero J. (2019). Programmed DNA Elimination of Germline Development Genes in Songbirds. Nat. Commun. 10, 5468. 10.1038/s41467-019-13427-4 31784533 PMC6884545

[B49] Knief U. Forstmeier W. Pei Y. Wolf J. Kempenaers B. (2020). A Test for Meiotic Drive in Hybrids between Australian and Timor Zebra Finches. Ecol. Evol. 10, 13464–13475. 10.1002/ece3.6951 33304552 PMC7713956

[B50] Koubová M. Pokorná M. J. Rovatsos M. Farkačová K. Altmanová M. Kratochvíl L. (2014). Sex Determination in Madagascar Geckos of the Genus *Paroedura* (Squamata: Gekkonidae): Are Differentiated Sex Chromosomes Indeed So Evolutionary Stable? Chromosome Res. 22, 441–452. 10.1007/s10577-014-9430-z 25056523

[B51] Kretschmer R. Ferguson-Smith M. De Oliveira E. (2018). Karyotype Evolution in Birds: From Conventional Staining to Chromosome Painting. Genes 9, 181. 10.3390/genes9040181 PMC5924523 29584697

[B52] Kretschmer R. Gunski R. J. Garnero A. D. V. Furo I. d. O. O'Brien P. C. M. Ferguson-Smith M. A. (2014). Molecular Cytogenetic Characterization of Multiple Intrachromosomal Rearrangements in Two Representatives of the Genus *Turdus* (Turdidae, Passeriformes). PLoS ONE 9, e103338. 10.1371/journal.pone.0103338 25058578 PMC4110018

[B53] Levan A. (1964). Nomenclature for Centromeric Position on Chromosomes. Hereditas 52, 201–220.

[B54] Malinovskaya L. P. Zadesenets K. S. Karamysheva T. V. Akberdina E. A. Kizilova E. A. Romanenko M. V. (2020). Germline-restricted Chromosome (GRC) in the Sand Martin and the Pale Martin (Hirundinidae, Aves): Synapsis, Recombination and Copy Number Variation. Sci. Rep. 10, 1058. 10.1038/s41598-020-58032-4 31974427 PMC6978364

[B55] Masabanda J. S. Burt D. W. O'Brien P. C. M. Vignal A. Fillon V. Walsh P. S. (2004). Molecular Cytogenetic Definition of the Chicken Genome: The First Complete Avian Karyotype. Genetics 166, 1367–1373. 10.1534/genetics.166.3.1367 15082555 PMC1470793

[B56] McLaughlin R. N. Malik H. S. (2017). Genetic Conflicts: the Usual Suspects and beyond. J. Exp. Biol. 220, 6–17. 10.1242/jeb.148148 28057823 PMC5278622

[B57] Minias P. Gutiérrez J. S. Dunn P. O. (2020). Avian Major Histocompatibility Complex Copy Number Variation Is Associated with Helminth Richness. Biol. Lett. 16, 20200194. 10.1098/rsbl.2020.0194 32634375 PMC7423054

[B58] Mittal O. P. Satija K. (1978). On the Somatic Chromosomes of *Saxicoloides Fulicata Combaiensis* (Latham). Proc. 65th Ind. Sci. Cong. Ahmedabad 3, 243.

[B59] Moens P. B. Heyting C. Dietrich A. J. van Raamsdonk W. Chen Q. (1987). Synaptonemal Complex Antigen Location and Conservation. J. Cel Biol. 105, 93–103. 10.1083/jcb.105.1.93 PMC2114919 2440900

[B60] Mořkovský L. Janoušek V. Reif J. Rídl J. Pačes J. Choleva L. (2018). Genomic Islands of Differentiation in Two Songbird Species Reveal Candidate Genes for Hybrid Female Sterility. Mol. Ecol. 27, 949–958. 10.1111/mec.14479 29319911 PMC5878113

[B61] Nanda I. Benisch P. Fetting D. Haaf T. Schmid M. (2011). Synteny Conservation of Chicken Macrochromosomes 1-10 in Different Avian Lineages Revealed by Cross-Species Chromosome Painting. Cytogenet. Genome Res. 132, 165–181. 10.1159/000322358 21099208

[B62] Nanda I. Schmid M. (1994). Localization of the Telomeric (TTAGGG)n Sequence in Chicken (*Gallus domesticus*) Chromosomes. Cytogenet. Cel Genet 65, 190–193. 10.1159/000133630 8222759

[B63] Nanda I. Schrama D. Feichtinger W. Haaf T. Schartl M. Schmid M. (2002). Distribution of Telomeric (TTAGGG)n Sequences in Avian Chromosomes. Chromosoma 111, 215–227. 10.1007/s00412-002-0206-4 12424522

[B64] Nguyen P. Sahara K. Yoshido A. Marec F. (2010). Evolutionary Dynamics of rDNA Clusters on Chromosomes of Moths and Butterflies (Lepidoptera). Genetica 138, 343–354. 10.1007/s10709-009-9424-5 19921441

[B65] Nishida C. Ishijima J. Kosaka A. Tanabe H. Habermann F. A. Griffin D. K. (2008). Characterization of Chromosome Structures of Falconinae (Falconidae, Falconiformes, Aves) by Chromosome Painting and Delineation of Chromosome Rearrangements during Their Differentiation. Chromosome Res. 16, 171–181. 10.1007/s10577-007-1210-6 18293111

[B66] Oliveira V. C. S. Altmanová M. Viana P. F. Ezaz T. Bertollo L. A. C. Ráb P. (2021). Revisiting the Karyotypes of Alligators and Caimans (Crocodylia, Alligatoridae) after a Half-century Delay: Bridging the gap in the Chromosomal Evolution of Reptiles. Cells 10, 1397. 10.3390/cells10061397 34198806 PMC8228166

[B67] Ortíz-Barrientos D. Reiland J. Hey J. Noor M. A. F. (2002). Recombination and the Divergence of Hybridizing Species. Genet. Mate Choice: Sex. Selection Sex. Isolation 116, 167–178. 10.1007/978-94-010-0265-3_2 12555775

[B68] Pardo-Manuel de Villena F. Sapienza C. (2001). Nonrandom Segregation during Meiosis: the Unfairness of Females. Mamm. Genome 12, 331–339. 10.1007/s003350040003 11331939

[B69] Peona V. Palacios-Gimenez O. M. Blommaert J. Liu J. Haryoko T. Jønsson K. A. (2021). The Avian W Chromosome Is a Refugium for Endogenous Retroviruses with Likely Effects on Female-Biased Mutational Load and Genetic Incompatibilities. Phil. Trans. R. Soc. B. 376, 20200186. 10.1098/rstb.2020.0186 34304594 PMC8310711

[B70] Perry G. H. Dominy N. J. Claw K. G. Lee A. S. Fiegler H. Redon R. (2007). Diet and the Evolution of Human Amylase Gene Copy Number Variation. Nat. Genet. 39, 1256–1260. 10.1038/ng2123 17828263 PMC2377015

[B71] Pertile M. D. Graham A. N. Choo K. H. A. Kalitsis P. (2009). Rapid Evolution of Mouse Y Centromere Repeat DNA Belies Recent Sequence Stability. Genome Res. 19, 2202–2213. 10.1101/gr.092080.109 19737860 PMC2792177

[B72] Peters A. H. F. M. Plug A. W. Van Vugt M. J. Boer P. d. (1997). A Drying-Down Technique for the Spreading of Mammalian Meiocytes from the Male and Female Germline. Chromosome Res. 5 (1), 66–68. 10.1023/a:1018445520117 9088645

[B73] Phadnis N. Orr H. A. (2009). A Single Gene Causes Both Male Sterility and Segregation Distortion in *Drosophila* Hybrids. Science 323, 376–379. 10.1126/science.1163934 19074311 PMC2628965

[B74] Pichugin A. M. Galkina S. A. Potekhin A. A. Punina E. O. Rautian M. S. Rodionov A. V. (2001). Estimation of the Minimal Size of Chicken *Gallus gallus domesticus* Microchromosomes via Pulsed-Field Electrophoresis. Russ. J. Genet. 37, 535–538. 10.1023/A:1016622816552 11436558

[B75] Pigozzi M. I. Solari A. J. (1998). Germ Cell Restriction and Regular Transmission of an Accessory Chromosome that Mimics a Sex Body in the Zebra Finch, *Taeniopygia guttata* . Chromosome Res. 6, 105–113. 10.1023/A:1009234912307 9543013

[B76] Pigozzi M. I. Solari A. J. (2005). The Germ-Line-Restricted Chromosome in the Zebra Finch: Recombination in Females and Elimination in Males. Chromosoma 114, 403–409. 10.1007/s00412-005-0025-5 16215738

[B77] Pokorná M. Rens W. Rovatsos M. Kratochvíl L. (2014). A ZZ/ZW Sex Chromosome System in the Thick-Tailed Gecko (*Underwoodisaurus milii*; Squamata: Gekkota: Carphodactylidae), a Member of the Ancient Gecko Lineage. Cytogenet. Genome Res. 142, 190–196. 10.1159/000358847 24603160

[B78] Reif J. Reifová R. Skoracka A. Kuczyński L. (2018). Competition-driven Niche Segregation on a Landscape Scale: Evidence for Escaping from Syntopy towards Allotopy in Two Coexisting Sibling Passerine Species. J. Anim. Ecol. 87, 774–789. 10.1111/1365-2656.12808 29430650

[B79] Reifová R. Kverek P. Reif J. (2011b). The First Record of a Female Hybrid between the Common Nightingale (*Luscinia megarhynchos*) and the Thrush Nightingale (*Luscinia luscinia*) in Nature. J. Ornithol 152, 1063–1068. 10.1007/s10336-011-0700-7

[B80] Reifová R. Reif J. Antczak M. Nachman M. W. (2011a). Ecological Character Displacement in the Face of Gene Flow: Evidence from Two Species of Nightingales. BMC Evol. Biol. 11, 138. 10.1186/1471-2148-11-138 21609448 PMC3121626

[B81] Rieseberg L. H. (2001). Chromosomal Rearrangements and Speciation. Trends Ecol. Evol. 16, 351–358. 10.1016/S0169-5347(01)02187-5 11403867

[B82] Rodionov A. V. Myakoshina Y. A. Chelysheva L. A. Solovei I. V. Gaginskaya E. R. (1992). Chiasmata in the Lampbrush Chromosomes of *Gallus gallus domesticus*: the Cytogenetic Study of Recombination Frequency and Linkage Map Lengths. Genetika 28, 53–63.

[B83] Rodrigues B. S. Kretschmer R. Gunski R. J. Garnero A. D. V. O'Brien P. C. M. Ferguson-Smith M. (2017). Chromosome Painting in Tyrant Flycatchers Confirms a Set of Inversions Shared by Oscines and Suboscines (Aves, Passeriformes). Cytogenet. Genome Res. 153, 205–212. 10.1159/000486975 29462803

[B84] Schartl M. Schmid M. Nanda I. (2016). Dynamics of Vertebrate Sex Chromosome Evolution: from Equal Size to Giants and Dwarfs. Chromosoma 125, 553–571. 10.1007/s00412-015-0569-y 26715206

[B85] Schindelin J. Arganda-Carreras I. Frise E. Kaynig V. Longair M. Pietzsch T. (2012). Fiji: an Open-Source Platform for Biological-Image Analysis. Nat. Methods 9, 676–682. 10.1038/nmeth.2019 22743772 PMC3855844

[B87] Skinner B. M. Al Mutery A. Smith D. Völker M. Hojjat N. Raja S. (2014). Global Patterns of Apparent Copy Number Variation in Birds Revealed by Cross-Species Comparative Genomic Hybridization. Chromosome Res. 22, 59–70. 10.1007/s10577-014-9405-0 24570127

[B88] Skinner B. M. Robertson L. B. Tempest H. G. Langley E. J. Ioannou D. Fowler K. E. (2009). Comparative Genomics in Chicken and Pekin Duck Using FISH Mapping and Microarray Analysis. BMC Genomics 10, 357. 10.1186/1471-2164-10-357 19656363 PMC2907691

[B89] Smith J. Bruley C. K. Paton I. R. Dunn I. Jones C. T. Windsor D. (2000). Differences in Gene Density on Chicken Macrochromosomes and Microchromosomes. Anim. Genet. 31, 96–103. 10.1046/j.1365-2052.2000.00565.x 10782207

[B90] Sottas C. Reif J. Kreisinger J. Schmiedová L. Sam K. Osiejuk T. S. (2020). Tracing the Early Steps of Competition-Driven Eco-Morphological Divergence in Two Sister Species of Passerines. Evol. Ecol. 34, 501–524. 10.1007/s10682-020-10050-4

[B91] Sottas C. Reif J. Kuczyński L. Reifová R. (2018). Interspecific Competition Promotes Habitat and Morphological Divergence in a Secondary Contact Zone between Two Hybridizing Songbirds. J. Evol. Biol. 31, 914–923. 10.1111/jeb.13275 29603471

[B92] Storchová R. Reif J. Nachman M. W. (2010). Female Heterogamety and Speciation: Reduced Introgression of the Z Chromosome between Two Species of Nightingales. Evolution 64, 456–471. 10.1111/j.1558-5646.2009.00841.x 19796142 PMC2911439

[B93] Sumner A. T. (1972). A Simple Technique for Demonstrating Centromeric Heterochromatin. Exp. Cel Res. 75, 304–306. 10.1016/0014-4827(72)90558-7 4117921

[B94] Symonová R. Sember A. Majtánová Z. Ráb P. (2015). “Characterization of Fish Genomes by GISH and CGH,” in Fish Cytogenetic Techniques. Editors Ozouf-Costaz C. Pisano E. Foresti F. de Almeida L. (Boca Paton: CRC Press), 118–131. 10.1201/b18534-17

[B95] Teixeira G. A. Aguiar H. J. a. C. Petitclerc F. Orivel J. Lopes D. M. Barros L. a. C. (2021). Evolutionary Insights into the Genomic Organization of Major Ribosomal DNA in Ant Chromosomes. Insect Mol. Biol. 30, 340–354. 10.1111/imb.12699 33586259

[B96] Torgasheva A. A. Malinovskaya L. P. Zadesenets K. S. Karamysheva T. V. Kizilova E. A. Akberdina E. A. (2019). Germline-restricted Chromosome (GRC) Is Widespread Among Songbirds. Proc. Natl. Acad. Sci. USA 116, 201817373. 10.1073/pnas.1817373116 PMC6575587 31036668

[B97] Udagawa T. (1955). Karyogram Studies in Birds VI. The Chromosomes of Five Species of the Turdidae. 日本動物学彙報 28 (4), 256–261.

[B98] Völker M. Backström N. Skinner B. M. Langley E. J. Bunzey S. K. Ellegren H. (2010). Copy Number Variation, Chromosome Rearrangement, and Their Association with Recombination during Avian Evolution. Genome Res. 20, 503–511. 10.1101/gr.103663.109 20357050 PMC2847753

[B99] Weissensteiner M. H. Bunikis I. Catalán A. Francoijs K.-J. Knief U. Heim W. (2020). Discovery and Population Genomics of Structural Variation in a Songbird Genus. Nat. Commun. 11, 3403. 10.1038/s41467-020-17195-4 32636372 PMC7341801

[B100] Wellenreuther M. Mérot C. Berdan E. Bernatchez L. (2019). Going beyond SNPs: The Role of Structural Genomic Variants in Adaptive Evolution and Species Diversification. Mol. Ecol. 28, 1203–1209. 10.1111/mec.15066 30834648

[B101] White M. J. D. (1978). Chain Processes in Chromosomal Speciation. Syst. Zoolog. 27, 285. 10.2307/2412880

[B102] Zhang G. Li B. Li B. Li C. Gilbert M. T. P. Jarvis E. D. (2014). Comparative Genomic Data of the Avian Phylogenomics Project. GigaSci 3, 26. 10.1186/2047-217X-3-26 PMC4322804 25671091

[B103] Zhang L. Reifová R. Halenková Z. Gompert Z. (2021). How Important Are Structural Variants for Speciation? Genes 12, 1084. 10.3390/genes12071084 34356100 PMC8305853

[B104] Zhang L. Sun T. Woldesellassie F. Xiao H. Tao Y. (2015). Sex Ratio Meiotic Drive as a Plausible Evolutionary Mechanism for Hybrid Male Sterility. Plos Genet. 11, e1005073. 10.1371/journal.pgen.1005073 25822261 PMC4379000

[B105] Zhou J. Lemos B. Dopman E. B. Hartl D. L. (2011). Copy-Number Variation: The Balance between Gene Dosage and Expression in *Drosophila melanogaster* . Genome Biol. Evol. 3, 1014–1024. 10.1093/gbe/evr023 21979154 PMC3227403

