# Supplementary material for: Comparison of Karyotypes in Two Hybridizing Passerine Species: Conserved Chromosomal Structure but Divergence in Centromeric Repeats
Source: Front Genet. 2021 Dec 6;12:768987. doi: 10.3389/fgene.2021.768987 (PMC8687609; doi:10.3389/fgene.2021.768987)
Supplement: Supplementary file 1 [file DataSheet1.docx]

***
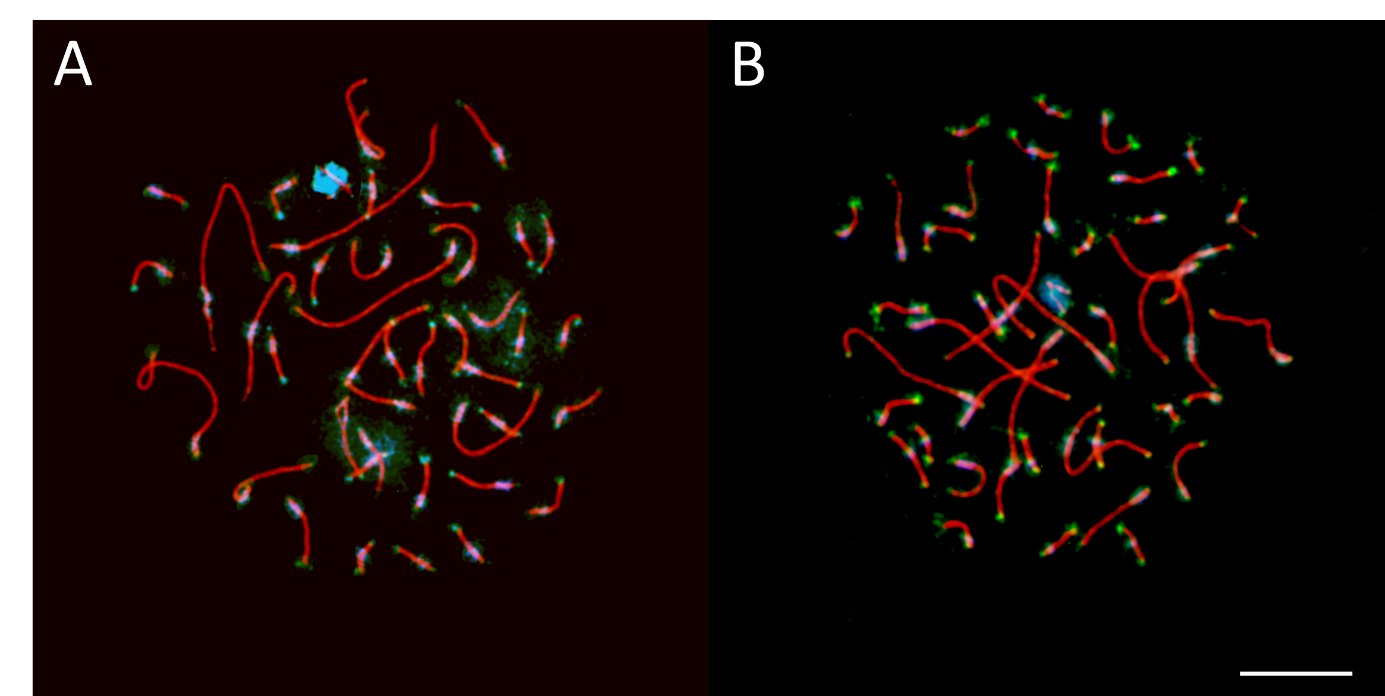
***

**Supplementary Figure 1.** Distribution of telomeric repeats (TTAGGG)n on meiotic chromosomes. Pachytene chromosomes of the common nightingale (L. megarhynchos) **(A)** and the thrush nightingale (L. luscinia) **(B)** immunostained with anti-SYCP3 antibody (red), the centromere protein CREST (blue) and telomeric (TTAGGG)_n_ probe (green). The germline restricted chromosome (GRC) is covered by the CREST signal along its entire length. Scalebar = 10 µm.

**Supplementary Table 1.** Sequence of the probe used for the FISH detection of 18S rDNA in nightingales.

| **Sequence name** | **Sequence** |
| --- | --- |
| *Anguis fragilis* 18S ribosomal RNA gene partial sequence | CCGCTTTGGTGACTCTTGATAACCTCGGGCCGATCGCACGCCCCCGTGGCGGCGACGACGCATTCGAATGTCTGCCCTATCGACTTTCGATGGTACTTTCTGTGCCTACCATGGTGACCACGGGTAACGGGGAATCAGGGTTCGATTCCGGAGAGGGAGCCTGAGAAACGGCTACCACATCCAAGGAAGGCAGCAGGCGCGCAAATTACCCACTCCCGACCCGGGGAGGTAGTGACGAAAAATAACAATACAGGACTCTTTCGAGGCCCTGTAATTGGAATGAGTACACTTTAAATCCTTTAACGAGGATCCATTGGAGGGCAAGTCTGGTGCCAGCAGCCGCGGTAATTCCAGCTCCAATAGCGTATCTTAAAGTTGCTGCAGTTAAAAAGCTCGTAGTTGGATCTTGGGATCGAGCTGGCGGTCCGCCGCGAGGCGAGCTACCGCCTGTCCCAGCCCCTGCCTCTCGGCGCTCCCTCGATGCTCTTCGCTGAGTGTCCCGGGGGTCCGAAGCGTTTACTTTGAAAAAATTAGAGTGTTCAAAGCAGGCCGGTCGCCGGAATACTCCAGCTAGGAATAATGGAATAGGACTCCGGTTCTATTTTGTTGGTTTTCGGAACTGGGGCCATGATTAAGAGGGACGGCCGGGGGCATTCGTATTGTGCCGCTAGAGGTGAAATTCTTGGACCGGCGCAAGACGAACCAGAGCGAAAGCATTTGCCAAGAATGTTTTCATTAATCAAGAACGAAAGTCGGAGGTTCGAAGACGATCAGATACCGTCGTAGTTCCGACCATAAACGATGCCGACTAGCGATCCGGCGGCGTTATTCCCATGACCCGCCGGGCAGCTTCCGGGAAACCAAAGTCTTTGGGTTCCGGGGGGAGTATGGTTGCAAAGCTGAAACTTAAAGGAATTGACGGAAGGGCACCACCAGGAGTGGAGCCTGCGGCTTAATTTGACTCAGCACGGGAAACCTCACCCGGCCCGGACACGGAAAGGATTGACAGATTGATAGCTCtTTCTCGATTCTGTGGGTGGTGGTGCATGGCCGTTCTTAGTTGGTGGAGCGATTTGTCTGGTTaATTCCGATaACGAACGAGACTCTGGCATGCTAACTAGTTaTGCGACCCCCGAGcGGTCGGCGTCCAACTTCTTAGAGGGACAAGTGGCGTTCAGCCACCCGAGATTGAGCAATAACAGGTCTGTGATGCCCTTAGATGTACGGGGCTGCACGCGCGCTACACTGACTGGCTCAGCGTGTGTCTACCCTACGCCGACAGGTGCGGGTAACCCGTTGAACCCCATTCGTGATGGGGATCGGGGATTGCAATTCTTCCCCATGAACGAGGAATTCCCAGTAAGTGCGGGTCATAAGCTCGCGTTGATTAAGTCCCTGCCCTTTGTACACACCGCCCGTCGCTACTACCGATTGGATGGTTTAGTGAGGCCCTCGG |

**Supplementary Table 2.** Size and morphology of chromosome bivalents during the pachytene stage. The averages of: the total length, the short arm (p) and the long arm (q) were calculated from 15 cells per species using the synaptonemal complex. Morphology classification is based on the arm ratio. m stands for metacentric, sm for submetacentric, a for acrocentric, and t for telocentric, with m/sm and a/t categories merged for microchromosomes due to the difficulty in distinguishing them on the smaller chromosomes. * represents the potential Z chromosome.

|  | **common nightingale** | | | | | **thrush nightingale** | | | | |
| --- | --- | --- | --- | --- | --- | --- | --- | --- | --- | --- |
|  | **average length ± sd (µm)** | | | **arm ratio** | **morpho-logy** | **average length ± sd (µm)** | | | **arm ratio** | **morpho- logy** |
| **SC** | **p** | **q** | **total** | **median** |  | **p** | **q** | **total** | **median** |  |
| **1** | 4,86 ± 1,35 | 24,65 ± 8,17 | 29,5 ± 8,61 | 4,69 | a | 4,52 ± 0,79 | 19,91 ± 4,55 | 24,44 ± 4,87 | 4,29 | a |
| **2** | 1,54 ± 0,38 | 23,6 ± 6,27 | 25,14 ± 6,54 | 15,68 | t | 0,99 ± 0,35 | 19,46 ± 2,86 | 20,45 ± 2,79 | 21,05 | t |
| **3** | 1,75 ± 0,99 | 21,9 ± 5,32 | 23,66 ± 5,63 | 13,92 | t | 1,26 ± 0,38 | 16,79 ± 2 | 18,04 ± 2,14 | 13,83 | t |
| **4*** | 1,37 ± 0,63 | 20,58 ± 4,53 | 21,95 ± 4,87 | 14,18 | t | 1,25 ± 0,35 | 16,03 ± 1,77 | 17,28 ± 1,77 | 12,06 | t |
| **5** | 5,01 ± 1,34 | 14,94 ± 3,93 | 19,93 ± 5,06 | 2,84 | sm | 4,53 ± 1,3 | 11,93 ± 2,07 | 16,46 ± 2,4 | 2,91 | sm |
| **6** | 6,86 ± 1,7 | 10,42 ± 2,38 | 17,36 ± 4,09 | 1,54 | m | 6,12 ± 1,22 | 9,28 ± 1,57 | 15,4 ± 1,62 | 1,36 | m |
| **7** | 1,47 ± 0,41 | 15,89 ± 3,82 | 17,32 ± 4,12 | 10,79 | t | 1,04 ± 0,32 | 12,88 ± 1,45 | 13,92 ± 1,45 | 12,55 | t |
| **8** | 1,54 ± 0,41 | 11,6 ± 2,47 | 13,14 ± 2,7 | 7,65 | t | 1,13 ± 0,44 | 9,64 ± 1,27 | 10,77 ± 1,16 | 9,01 | t |
| **9** | 1,38 ± 0,45 | 10,98 ± 2,54 | 12,37 ± 2,58 | 7,49 | t | 0,93 ± 0,5 | 9 ± 0,95 | 9,92 ± 0,73 | 9,3 | t |
| **10** | 1,6 ± 0,43 | 9,57 ± 2,2 | 11,17 ± 2,37 | 5,95 | a | 0,95 ± 0,27 | 8,43 ± 0,72 | 9,37 ± 0,72 | 9,12 | t |
| **11** | 1,42 ± 0,59 | 8,57 ± 1,92 | 9,99 ± 2,22 | 6,63 | a/t | 1,34 ± 0,49 | 7,32 ± 1,04 | 8,66 ± 0,73 | 4,74 | a/t |
| **12** | 1,46 ± 0,36 | 7,41 ± 1,58 | 8,88 ± 1,76 | 4,43 | a/t | 1,31 ± 0,57 | 6,86 ± 0,51 | 8,18 ± 0,64 | 5,88 | a/t |
| **13** | 1,64 ± 0,61 | 6,65 ± 1,5 | 8,27 ± 1,57 | 4,96 | a/t | 1,12 ± 0,47 | 6,43 ± 0,84 | 7,54 ± 0,7 | 6,56 | a/t |
| **14** | 1,55 ± 0,57 | 6,54 ± 1,69 | 8,09 ± 1,59 | 4,61 | a/t | 1,01 ± 0,28 | 6,2 ± 0,57 | 7,2 ± 0,64 | 6,09 | a/t |
| **15** | 1,45 ± 0,38 | 6,48 ± 1,51 | 7,93 ± 1,59 | 4,56 | a/t | 1,17 ± 0,53 | 5,73 ± 0,91 | 6,9 ± 0,62 | 5,68 | a/t |
| **16** | 1,59 ± 0,82 | 6,26 ± 1,17 | 7,85 ± 1,61 | 4,46 | a/t | 1,18 ± 0,61 | 5,35 ± 0,5 | 6,52 ± 0,43 | 5,77 | a/t |
| **17** | 1,56 ± 0,64 | 6,16 ± 1,26 | 7,72 ± 1,56 | 4,83 | a/t | 1,02 ± 0,38 | 5,25 ± 0,68 | 6,26 ± 0,42 | 6,17 | a/t |
| **18** | 1,33 ± 0,36 | 6,19 ± 1,38 | 7,52 ± 1,52 | 4,87 | a/t | 0,83 ± 0,45 | 5,14 ± 0,73 | 5,97 ± 0,46 | 6,91 | a/t |
| **19** | 1,43 ± 0,56 | 5,94 ± 1,71 | 7,37 ± 1,47 | 4,41 | a/t | 0,98 ± 0,6 | 4,8 ± 0,65 | 5,77 ± 0,39 | 6,19 | a/t |
| **20** | 1,66 ± 0,85 | 5,53 ± 1,13 | 7,19 ± 1,43 | 3,85 | a/t | 0,71 ± 0,5 | 4,82 ± 0,63 | 5,53 ± 0,33 | 8,51 | a/t |
| **21** | 1,74 ± 0,75 | 5,23 ± 1,6 | 6,97 ± 1,38 | 3,52 | a/t | 1,84 ± 0,6 | 3,45 ± 0,63 | 5,29 ± 0,31 | 1,59 | sm/m |
| **22** | 1,62 ± 0,74 | 5,14 ± 1,07 | 6,76 ± 1,27 | 3,76 | a/t | 0,55 ± 0,27 | 4,63 ± 0,45 | 5,19 ± 0,28 | 9,56 | a/t |
| **23** | 1,54 ± 0,54 | 5,04 ± 1,21 | 6,58 ± 1,2 | 3,12 | a/t | 1,28 ± 0,8 | 3,71 ± 0,82 | 4,98 ± 0,25 | 4,08 | a/t |
| **24** | 1,41 ± 0,52 | 4,98 ± 0,94 | 6,39 ± 1,18 | 3,55 | a/t | 0,89 ± 0,63 | 3,99 ± 0,67 | 4,86 ± 0,33 | 6,25 | a/t |
| **25** | 1,14 ± 0,39 | 5,02 ± 0,95 | 6,16 ± 1,16 | 4,37 | a/t | 1,13 ± 0,76 | 3,54 ± 0,99 | 4,67 ± 0,32 | 5,9 | a/t |
|  | **common nightingale** | | | | | **thrush nightingale** | | | | |
|  | **average length ± sd (µm)** | | | **arm ratio** | **morpho-logy** | **average length ± sd (µm)** | | | **arm ratio** | **morpho- logy** |
| **SC** | **p** | **q** | **total** | **median** |  | **p** | **q** | **total** | **median** |  |
| **26** | 1,46 ± 0,34 | 4,3 ± 0,89 | 5,76 ± 1,09 | 3,07 | a/t | 0,65 ± 0,27 | 3,76 ± 0,21 | 4,41 ± 0,26 | 6,4 | a/t |
| **27** | 1,75 ± 0,84 | 3,79 ± 0,73 | 5,53 ± 0,99 | 2,13 | sm/m | 1,61 ± 0,45 | 2,59 ± 0,52 | 4,21 ± 0,38 | 1,5 | sm/m |
| **28** | 1,37 ± 0,52 | 4 ± 0,93 | 5,36 ± 0,91 | 2,79 | sm/m | 0,82 ± 0,36 | 3,27 ± 0,39 | 4,09 ± 0,23 | 4,74 | a/t |
| **29** | 1,43 ± 0,55 | 3,76 ± 0,72 | 5,18 ± 0,84 | 3,06 | a/t | 1,09 ± 0,55 | 2,82 ± 0,56 | 3,91 ± 0,25 | 3,17 | a/t |
| **30** | 1,41 ± 0,51 | 3,63 ± 0,72 | 5,05 ± 0,86 | 2,05 | sm/m | 1,12 ± 0,49 | 2,66 ± 0,49 | 3,78 ± 0,21 | 2,41 | sm/m |
| **31** | 1,55 ± 0,64 | 3,37 ± 0,78 | 4,92 ± 0,84 | 2,31 | sm/m | 1,2 ± 0,47 | 2,48 ± 0,47 | 3,68 ± 0,22 | 1,88 | sm/m |
| **32** | 1,55 ± 0,48 | 3,27 ± 0,73 | 4,82 ± 0,85 | 2,05 | sm/m | 1,19 ± 0,54 | 2,41 ± 0,49 | 3,6 ± 0,25 | 1,85 | sm/m |
| **33** | 1,47 ± 0,41 | 3,24 ± 0,81 | 4,71 ± 0,85 | 2,08 | sm/m | 1,05 ± 0,41 | 2,46 ± 0,53 | 3,51 ± 0,23 | 2,52 | sm/m |
| **34** | 1,49 ± 0,55 | 3,15 ± 0,81 | 4,65 ± 0,84 | 1,78 | sm/m | 0,94 ± 0,52 | 2,46 ± 0,5 | 3,39 ± 0,21 | 2,65 | sm/m |
| **35** | 1,43 ± 0,5 | 3,13 ± 0,72 | 4,57 ± 0,82 | 2,12 | sm/m | 0,89 ± 0,37 | 2,4 ± 0,37 | 3,28 ± 0,22 | 3,27 | a/t |
| **36** | 1,48 ± 0,45 | 3,04 ± 0,77 | 4,52 ± 0,82 | 2,12 | sm/m | 0,74 ± 0,52 | 2,38 ± 0,49 | 3,12 ± 0,27 | 4,34 | a/t |
| **37** | 1,36 ± 0,34 | 3,06 ± 0,69 | 4,42 ± 0,78 | 2,21 | sm/m | 1,05 ± 0,28 | 1,9 ± 0,35 | 2,94 ± 0,3 | 1,71 | sm/m |
| **38** | 1,56 ± 0,42 | 2,78 ± 0,71 | 4,34 ± 0,76 | 1,76 | sm/m | 0,94 ± 0,3 | 1,87 ± 0,32 | 2,8 ± 0,22 | 2,08 | sm/m |
| **39** | 1,51 ± 0,47 | 2,73 ± 0,56 | 4,24 ± 0,72 | 1,79 | sm/m | 0,97 ± 0,26 | 1,76 ± 0,33 | 2,73 ± 0,23 | 1,74 | sm/m |
| **40** | 1,59 ± 0,5 | 2,57 ± 0,58 | 4,15 ± 0,7 | 1,57 | sm/m | 0,92 ± 0,32 | 1,74 ± 0,4 | 2,66 ± 0,22 | 1,53 | sm/m |
| **41** | 1,49 ± 0,4 | 2,48 ± 0,5 | 3,97 ± 0,67 | 1,45 | sm/m | 0,93 ± 0,38 | 1,63 ± 0,34 | 2,56 ± 0,18 | 1,4 | sm/m |
| **42** | 1,52 ± 0,34 | 2,23 ± 0,45 | 3,75 ± 0,68 | 1,41 | sm/m | 0,76 ± 0,34 | 1,63 ± 0,34 | 2,4 ± 0,19 | 1,76 | sm/m |
| **GRC** | 0,55 ± 0,23 | 8,1 ± 1,27 | 8,64 ± 1,29 | 17,29 | a/t | 0,63 ± 0,48 | 5,32 ± 1,03 | 5,96 ± 1,01 | 9,12 | a/t |

**Supplementary Table 3.** Signal intensities of the common nightingale (*L. megarhynchos*) DNA probe (green) and the thrush nightingale (*L. luscinia*) DNA probe (red) at centromeric regions from the interspecific comparative genomic hybridization (CGH) experiment. DNA probes were hybridized to the common nightingale and the thrush nightingale metaphases. Signal intensities are shown after normalization for the nine macrochromosomes and sex chromosomes.

|  | **common nightingale** | | | | | **thrush nightingale** | | | | |
| --- | --- | --- | --- | --- | --- | --- | --- | --- | --- | --- |
| **Chr** | **red average** | **green average** | **ratio** | **log2** | **sd** | **red average** | **green average** | **ratio** | **log2** | **sd** |
| **1** | 0.019 | 0.019 | 1.023 | 0.033 | 0.145 | 0.032 | 0.028 | 1.147 | 0.198 | 0.087 |
| **2** | 0.022 | 0.025 | 0.866 | -0.207 | 0.061 | 0.026 | 0.022 | 1.154 | 0.207 | 0.061 |
| **3** | 0.019 | 0.026 | 0.850 | -0.234 | 0.139 | 0.028 | 0.025 | 1.104 | 0.143 | 0.099 |
| **4** | 0.021 | 0.023 | 0.919 | -0.121 | 0.105 | 0.019 | 0.019 | 1.004 | 0.005 | 0.187 |
| **5** | 0.021 | 0.020 | 1.135 | 0.183 | 0.179 | 0.030 | 0.026 | 1.130 | 0.177 | 0.081 |
| **6** | 0.023 | 0.027 | 0.825 | -0.278 | 0.124 | 0.026 | 0.021 | 1.236 | 0.305 | 0.123 |
| **7** | 0.029 | 0.035 | 0.885 | -0.176 | 0.225 | 0.028 | 0.023 | 1.161 | 0.215 | 0.164 |
| **8** | 0.025 | 0.025 | 0.841 | -0.249 | 0.080 | 0.031 | 0.031 | 1.137 | 0.186 | 0.107 |
| **9** | 0.023 | 0.025 | 0.940 | -0.089 | 0.156 | 0.027 | 0.023 | 1.166 | 0.221 | 0.140 |
| **Z** | 0.020 | 0.024 | 0.800 | -0.323 | 0.150 | 0.025 | 0.029 | 0.863 | -0.212 | 0.102 |
| **W** | 0.040 | 0.035 | 1.139 | 0.187 | 0.128 | 0.031 | 0.029 | 1.059 | 0.083 | 0.011 |
